# Non-alcoholic Fatty Liver Disease Is Associated With Cardiovascular Outcomes in Subjects With Prediabetes and Diabetes: A Prospective Community-Based Cohort Study

**DOI:** 10.3389/fcvm.2022.889597

**Published:** 2022-04-26

**Authors:** Qi-Rui Song, Shuo-Lin Liu, Ya-Guang Bi, Shuo-Hua Chen, Shou-Ling Wu, Jun Cai

**Affiliations:** ^1^Hypertension Center, Fuwai Hospital, State Key Laboratory of Cardiovascular Disease of China, National Center for Cardiovascular Diseases of China, Chinese Academy of Medical Sciences and Peking Union Medical College, Beijing, China; ^2^Department of Cardiology, Zhongshan Hospital, Fudan University, Shanghai, China; ^3^Shanghai Institute of Cardiovascular Diseases, Shanghai, China; ^4^Department of Cardiology, Kailuan General Hospital, Tangshan, China

**Keywords:** non-alcoholic fatty liver disease, cardiovascular disease, diabetes mellitus, coronary artery disease, prognosis

## Abstract

**Background:**

There have been no studies of the effect of non-alcoholic fatty liver disease (NAFLD) on cardiovascular events (CVEs) in patients with pre-diabetes (pre-DM), and diabetes mellitus (DM). We performed a community-based cohort study to evaluate the relationship between NAFLD and CVEs in patients with glucose metabolism disorder.

**Methods:**

We enrolled 71,852 participants from the Kailuan study who had not experienced CVEs, after excluding alcohol abuse and other liver diseases. NAFLD was assessed using abdominal ultrasonography. Besides, participants were categorized by glucose metabolism status [normal glucose regulation (NGR), pre-DM, and DM]. All subjects were followed up for the occurrence of CVEs.

**Results:**

During a median of 13.01 (0.64) years of follow-up, 6,037 CVEs occurred. NAFLD was present in 22,525 (31.3%), and compared with participants without NAFLD, those with NAFLD had a 12.3% [95% confidence interval (CI) 1.059–1.191, *P* < 0.001] higher risk of CVEs, after adjustment for potential confounders. The hazard ratios for patients with mild, moderate, and severe NAFLD were 1.104 (95% CI 1.035–1.179, *P* < 0.001), 1.149 (95% CI 1.055–1.251, *P* < 0.001), and 1.235 (95% CI 1.059–1.441, *P* < 0.001), respectively. Moreover, participants with pre-DM plus NAFLD and participants with DM plus NAFLD had 1.267-fold (95% CI 1.151–1.395, *P* < 0.001) and 1.829-fold (95% CI 1.666–2.008, *P* < 0.001) higher risks of CVEs, respectively, compared with those with NGR and no NAFLD. The addition of the combination of NAFLD and glucose metabolism status to the crude Cox model increased the C-statistic by 0.0066 (0.0053–0.0080, *P* < 0.001).

**Conclusions:**

NAFLD is associated with higher risks of CVEs. Moreover, NAFLD is an independent predictor of CVEs in patients with pre-DM and DM, suggesting that NAFLD may provide greater risk predictive value for patients with glucose metabolism disorder.

## Background

Non-alcoholic fatty liver disease (NAFLD) is becoming the commonest cause of chronic liver disease, affecting ~25% of the general population worldwide. In China, the prevalence of NAFLD has doubled over the past 20 years (1).

NAFLD involves the pathological accumulation of fat in the liver and is associated with the pathogenesis of multiple metabolic disorders, including hyperlipidemia, obesity, the metabolic syndrome, insulin resistance, and diabetes. These conditions may interact synergistically to increase the risks of hepatic and extrahepatic complications and mortality (2). The findings of a number of epidemiological studies imply that NAFLD is an independent predictor of subclinical cardiovascular diseases, such as artery endothelial dysfunction, thickening of the carotid artery wall, coronary artery calcification, and the progression of atherosclerosis (3–6). Moreover, NAFLD is also associated with coronary artery stenosis and the need for coronary artery intervention (7). However, previous studies have generated contrasting data regarding the relationship between NAFLD and long-term clinical cardiovascular outcomes (7–10).

NAFLD and glucose metabolism disorders are common metabolic diseases that regularly coexist and each affects the progression of the other. Individuals with NAFLD are predisposed toward a deterioration of insulin sensitivity (11), which results in a two-fold higher incidence of type 2 diabetes mellitus (T2DM) (12). Furthermore, NAFLD substantially increases the risk of developing cardiovascular complications of diabetes (13). Interestingly, the presence of prediabetes (pre-DM) is not an independent risk factor for cardiovascular events (CVEs), but it does increase the risk of a CVE in combination with other metabolic disorders (14, 15). However, it is unknown whether NAFLD is associated with poorer cardiovascular outcomes in patients with prediabetes.

We aimed to determine the effect of NAFLD on cardiovascular outcomes in a large cohort of Chinese people derived from the general population, who had normal glucose regulation (NGR), pre-diabetes (pre-DM), or diabetes mellitus (DM).

## Materials and Methods

### Study Design and Population

The data were extracted from that collected during the Kailuan study (trial registration number, ChiCTR-TNC-1100148; trial registration site, http://www.chictr.org.cn/index.aspx). This is a community-based prospective cohort study being performed in Kailuan, Tangshan City, northern China. Detailed information regarding the study design and procedures of the Kailuan study has been published previously (16). Briefly, a total of 101,510 participants (81,110 men and 20,400 women, aged 18–98 years) were recruited for the initial survey, and they completed questionnaires and underwent physical examinations and laboratory testing in the 11 hospitals in the Kailuan community between June 2006 and October 2007. Re-examinations were conducted every 2 years until December 31, 2019 or death.

**Figure 1:**
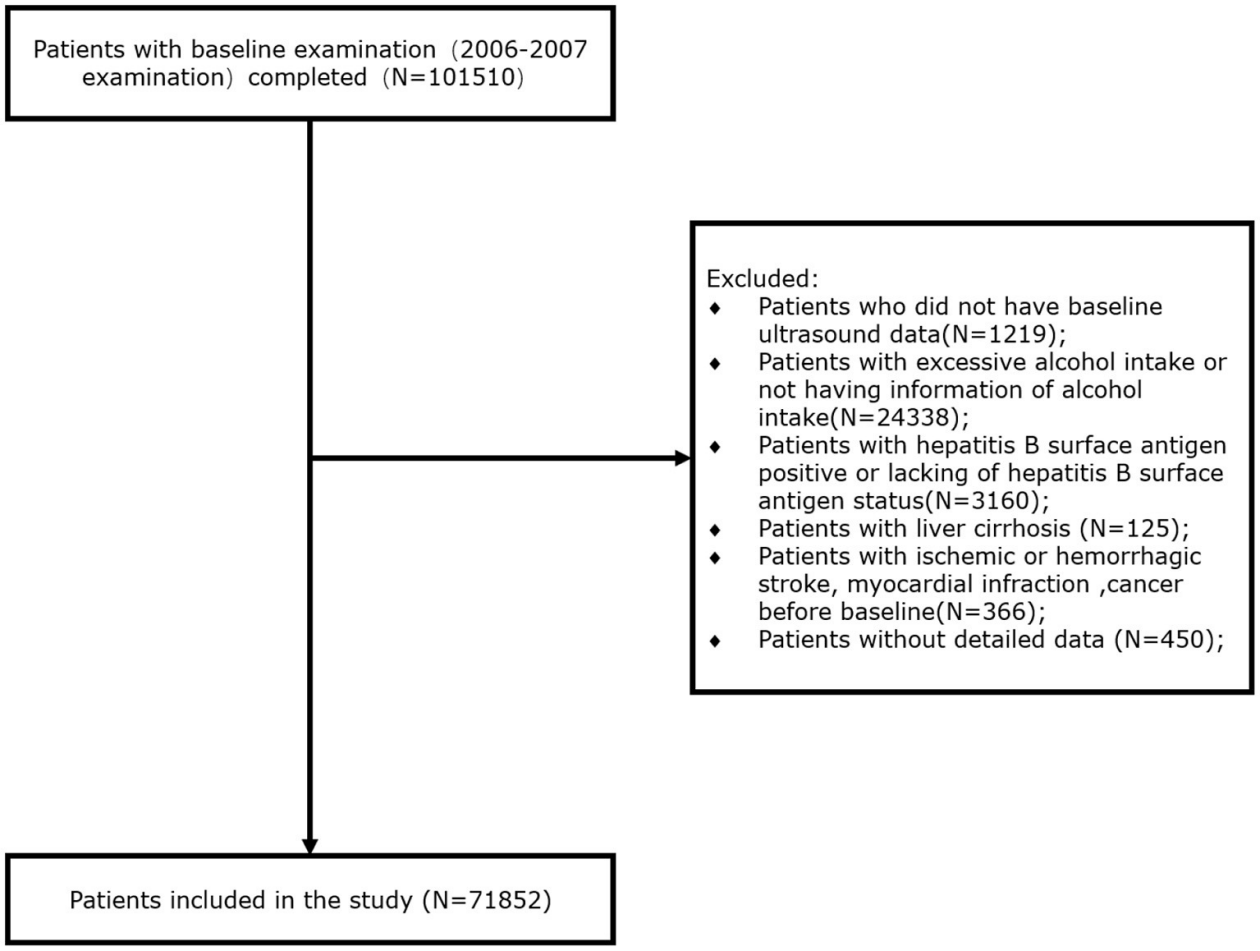
Flowchart of the study.

Of the participants recruited for the initial survey, we excluded 1,219 for whom baseline ultrasonographic data were not available, 24,338 for whom information regarding alcohol intake was not available or who consumed an excessive amount of alcohol (defined as an alcohol intake ≥30 g/day for men and ≥20 g/day for women), 3,160 who were positive for hepatitis B surface antigen (HBsAg) or for whom this information was not available, 125 with liver cirrhosis, 366 who had previously experienced ischemic or hemorrhagic stroke, myocardial infarction, or cancer, and 450 for whom detailed data were not available. Thus, 71,852 participants who were recruited at baseline were included in the present study (Figure 1).

### Assessment of NAFLD

Experienced radiologists who were blinded to the clinical presentation and laboratory findings of each participant performed abdominal ultrasonography. A high-resolution B-mode topographical ultrasound system with a 3.5 MHz probe (Acuson X300, Siemens, Munich, Germany) was used.

NAFLD was diagnosed on the basis of the presence of at least two of the following abnormal findings on abdominal ultrasonography, according to the criteria for the assessment and management of NAFLD in China (17): (1) increased echogenicity of the liver near-field region with deep attenuation of the ultrasound signal; (2) hyperechogenicity of liver tissue (“bright liver”), compared with the echogenicity of the kidney cortex; and (3) vascular blurring. NAFLD was diagnosed in the presence of fatty liver and in the absence of excessive alcohol consumption or other causes of chronic liver disease (18).

The severity of NAFLD was defined ultrasonographically, as previously described (19), as mild (diffuse increase in fine echoes from the liver parenchyma), moderate (diffuse increase in fine echoes, with impaired visualization of the intrahepatic vessel borders and diaphragm), or severe (diffuse increase in fine echoes, with no visualization possible of the intrahepatic vessel borders or diaphragm).

### Definitions of Diabetes and Prediabetes

Blood samples were obtained from the anterior elbow of the participants under fasting conditions (at least 8 h) in the morning. Fasting blood glucose (FBG) concentration was measured using the hexokinase/glucose-6-phosphate dehydrogenase method (Mind Bioengineering Co. Ltd, Shanghai, China). DM was diagnosed if participants had an FBG of ≥7.00 mmol/L, self-reported a physician diagnosis, or self-reported the use of antidiabetic medication. Pre-DM was defined using an FBG of 5.60–6.99 mmol/L for participants who were not taking antidiabetic medication. Participants without pre-DM or DM were defined as having normal NGR.

### Definitions of Outcomes

The primary outcome of the present study was an incident CVE, including myocardial infarction (MI), hemorrhagic stroke (HS), and ischemic stroke (IS). The CVEs were identified by ICD 10th edition codes, as described previously (16). Information regarding incident CVEs was gathered at biennial interviews and from annual discharge lists, which were reviewed and adjudicated by a committee consisting of three experienced blinded physicians from the 11 Kailuan hospitals. Information regarding mortality was collected from the provincial vital statistics offices and reviewed by clinicians. In line with the World Health Organization's Multinational Monitoring of Trends and Determinants in Cardiovascular Disease (MONICA) criteria (20), MI was diagnosed on the basis of clinical symptoms, high circulating cardiac enzyme activities, or biomarker concentrations, as well as electrocardiography. Stroke was diagnosed according to the World Health Organization criteria (21) on the basis of neurological signs, clinical symptoms, neurological images (computed tomography or magnetic resonance imaging), and other diagnostic reports, as described previously (16).

### Assessments of Covariates

Plasma alanine aminotransferase (ALT) activity and the plasma total bilirubin (TBIL), creatinine (CR), uric acid (UA), triglyceride (TG), total cholesterol (TC), high-density lipoprotein-cholesterol (HDL-C), and low-density lipoprotein-cholesterol (LDL-C) concentrations were measured in the central laboratory of Kailuan hospital using an auto-analyzer (Hitachi 747; Hitachi, Tokyo, Japan). Hepatitis B surface antigen (HBsAg) was assayed using an enzyme-linked immunosorbent assay (Shanghai Kehua Bio-Engineering, Shanghai, China). Plasma high-sensitivity C-reactive protein concentration was measured using a high-sensitivity particle-enhanced immunonephelometric assay (Cias Latex CRP-H, Kanto Chemical Co Inc, Tokyo, Japan). Estimated glomerular filtration rate (eGFR) was calculated using the Chronic Kidney Disease Epidemiology Collaboration creatinine equation (22). Hyperlipidemia was defined using a compatible medical history, a fasting total cholesterol (TC) concentration ≥5.18 mmol/L, or a triglyceride (TG) concentration ≥1.7 mmol/L.

Blood pressure (BP) was measured by well-trained operators between 07:00 and 09:00 on the day of the physical examination. Smoking, caffeine consumption, and physical activity were prohibited for 30 min prior to the measurement and the participants were seated quietly for at least 5 min beforehand. Mercury sphygmomanometers were used to measure BP three times at intervals of 1–2 min, and the mean value was obtained. To avoid measurement bias, the sphygmomanometers were regularly checked and calibrated. The heart rate, height, and weight of the participants were measured by trained field workers during the surveys. Body mass index (BMI) was calculated as weight (kg) divided by the square of height (m^2^).

Information regarding sociodemographic parameters (e.g., birth date and sex), lifestyle (e.g., smoking status, alcohol consumption, and physical activity) and previous medical history (e.g., hypertension, diabetes, and the use of antihypertensive agents) was collected by trained interviewers using questionnaires, as described previously (16). Smokers were defined as participants who had a history of smoking or who were current smokers, and those who had a history of alcohol consumption or who currently consumed alcohol were defined asdrinkers. Physical activity was defined as engaging in aerobic exercise ≥3 times/week, for ≥30 min/session.

### Statistical Analysis

Statistical analyses were performed using SAS version 9.4 (SAS Institute, Inc, Cary, NC, USA) and R, version 3.5.2 (R Foundation for Statistical Computing, Vienna, Austria). Continuous variables are presented as mean ± standard deviation (SD) or median with interquartile range, and categorical variables are described as number or percentage. The Kolmogorov-Smirnov test was used to identify the distributions of the variables.

One-way ANOVA, the Kruskal-Wallis test or the chi-square test was used to compare the data according to the glucose control status of the participants, as appropriate. The event-free survival rates of the groups were determined using the Kaplan-Meier method and compared using the log-rank test. Univariate and multivariate cox proportional hazard regression models were used to calculate hazard ratios (HRs) and 95% confidence intervals (CIs) for incident cardiovascular disease for each group. Model 1 was unadjusted. Model 2 was adjusted for age, sex, BMI (≥30, 25–29.9, 18.5–24.9, or <18.5 kg/m^2^), physical activity (<3 or ≥3 times/week), and smoking status (never a smoker or a smoker). Model 3 was further adjusted for hyperlipidemia, the use of lipid-lowering medication, HDL-C, systolic blood pressure (SBP), high-sensitivity C-reactive protein (hsCRP), and eGFR. We conducted trend tests in the Cox proportional hazard regression models, with the median value of each group being included as a continuous variable.

We calculated the C-statistic and ΔC-statistic to evaluate the efficiency of the models and the incremental value of adding the combination of NAFLD and glucose control status to the original model. Sensitivity analyses were performed by excluding all the participants who experienced a CVE within the first year of follow-up to assess the robustness of the results. All the statistical tests were two-sided and *P* < 0.05 was considered to represent statistical significance.

## Results

### Baseline Characteristics of the Participants

The 71,852 participants were allocated to the NGR (*n* = 50,820), pre-DM (13,990), and DM (7,042) groups and the baseline characteristics of the sample are shown in Table 1. Participants with pre-DM or DM were more likely to be older; to have a large waist circumference, high BMI, high blood pressure, and high FBG; to be smokers and drinkers; to have high lipid (TC, LDL-C, and TG), creatinine, TBIL, and HsCRP concentrations; to have high AST activity; and to have low eGFR (all *P* < 0.001) than the NGR group. Moreover, the percentages of participants with hypertension or hyperlipidemia, and of those taking antihypertensive or antidiabetic medication, increased from the NGR to the pre-DM and the DM group (*P* < 0.001).

**Table 1:** Clinical characteristics of the participants, classified according to their glucose metabolism status.

**Variables**	**Total (*n* = 71,852)**	**NGR (*n* = 50,820)**	**Pre-DM (*n* = 13,990)**	**DM (*n* = 7,042)**	***P-*value**
Age (years)	51.83 ± 12.72	50.91 ± 13.06	52.58 ± 11.71	55.96 ± 10.58	<0.001
Male, *n* (%)	53,740 (75.75)	36,859 (72.53)	11,349 (81.12)	5,532 (78.56)	<0.001
Waist circumference (cm)	86.78 ± 9.91	85.97 ± 9.86	87.85 ± 9.80	90.52 ± 9.32	<0.001
Body mass index, (kg/m^2^)	25.07 ± 3.52	24.76 ± 3.48	25.63 ± 3.49	26.23 ± 3.45	<0.001
Hypertension, *n* (%)	31,964 (44.49)	20,387 (40.12)	7,072 (50.55)	4,505 (63.97)	<0.001
Diabetes, *n* (%)	7,042 (9.80)	0 (0)	0 (0)	7,042 (100)	<0.001
Hyperlipidemia, *n* (%)	24,834 (34.56)	15,778 (31.05)	5,325 (38.06)	3731 (52.98)	<0.001
Physical activity ≥3 times/week, *n* (%)	10,401 (14.48)	6,975 (13.72)	2,090 (14.94)	1,336 (18.97	<0.001
Current or previous smoking, *n* (%)	21,181 (29.48)	14,513 (28.56)	4,513 (32.26)	2,155 (30.60)	<0.001
Current or previous drinking, *n* (%)	17,616 (24.52)	12,041 (23.69)	3,869 (19.47)	1,706 (9.80)	<0.001
Systolic blood pressure (mmHg)	130.84 ± 21.17	128.74 ± 20.61	133.91 ± 21.21	139.82 ± 21.94	<0.001
Diastolic blood pressure (mmHg)	83.30 ± 11.70	82.40 ± 11.54	85.06 ± 11.69	86.29 ± 11.96	<0.001
**Laboratory findings**					
Fasting blood pressure, (mmol/L)	5.49 ± 1.73	4.80 ± 0.48	6.04 ± 0.36	9.37 ± 3.10	<0.001
Creatinine (mg/dL)	91.94 ± 30.54	91.84 ± 28.89	90.75 ± 31.13	76.42 ± 20.01	<0.001
eGFR (ml/min/1.73 m^2^)	80.35 ± 19.58	80.43 ± 19.25	82.08 ± 20.27	94.64 ± 36.53	<0.001
TG (mmol/L)	1.27 (0.90–1.91)	1.21 (0.86–1.78)	1.38 (0.99–2.08)	1.66 (1.15–2.56)	<0.001
TC (mmol/L)	4.93 ± 1.14	4.86 ± 1.11	5.06 ± 1.13	5.21 ± 1.32	<0.001
HDL-C (mmol/L)	1.52 ± 0.35	1.52 ± 0.35	1.50 ± 0.34	1.52 ± 0.36	<0.001
LDL-C (mmol/L)	2.33 ± 0.87	2.27 ± 0.85	2.49 ± 0.86	2.43 ± 0.96	<0.001
HsCRP (mg/L)	0.80 (0.30–2.12)	0.72 (0.27–2.00)	0.88 (0.33–2.19)	1.20 (0.49–3.08)	<0.001
ALT (U/L)	18.00 (13.00–24.00)	18.00 (12.00–24.00)	18.00 (13.00–25.00)	19.00 (14.00–26.00)	<0.001
TBIL (μmol/L)	12.77 ± 4.84	12.85 ± 4.88	12.45 ± 4.60	12.86 ± 5.03	<0.001
**Medications**					
Antihypertensive medication, *n* (%)	8,136 (11.32)	4,852 (9.55)	1,657 (11.84)	1,626 (23.09)	<0.001
Antidiabetic medication, *n* (%)	1,973 (2.75)	0 (0)	0 (0)	1,973 (28.02)	<0.001
Lipid-lowering medication, *n* (%)	728 (1.01)	383 (0.75)	119 (0.85)	226 (3.21)	<0.001

*eGFR, estimated glomerular filtration rate; TG, triglyceride; TC, total cholesterol; HDL-C, high-density lipoprotein cholesterol; LDL-C, low-density lipoprotein cholesterol; hsCRP, high-sensitivity C-reactive protein; ALT, alanine aminotransferase; TBIL, total bilirubin*.

### Relationships Between NAFLD and Cardiovascular Outcomes

**Figure 2:**
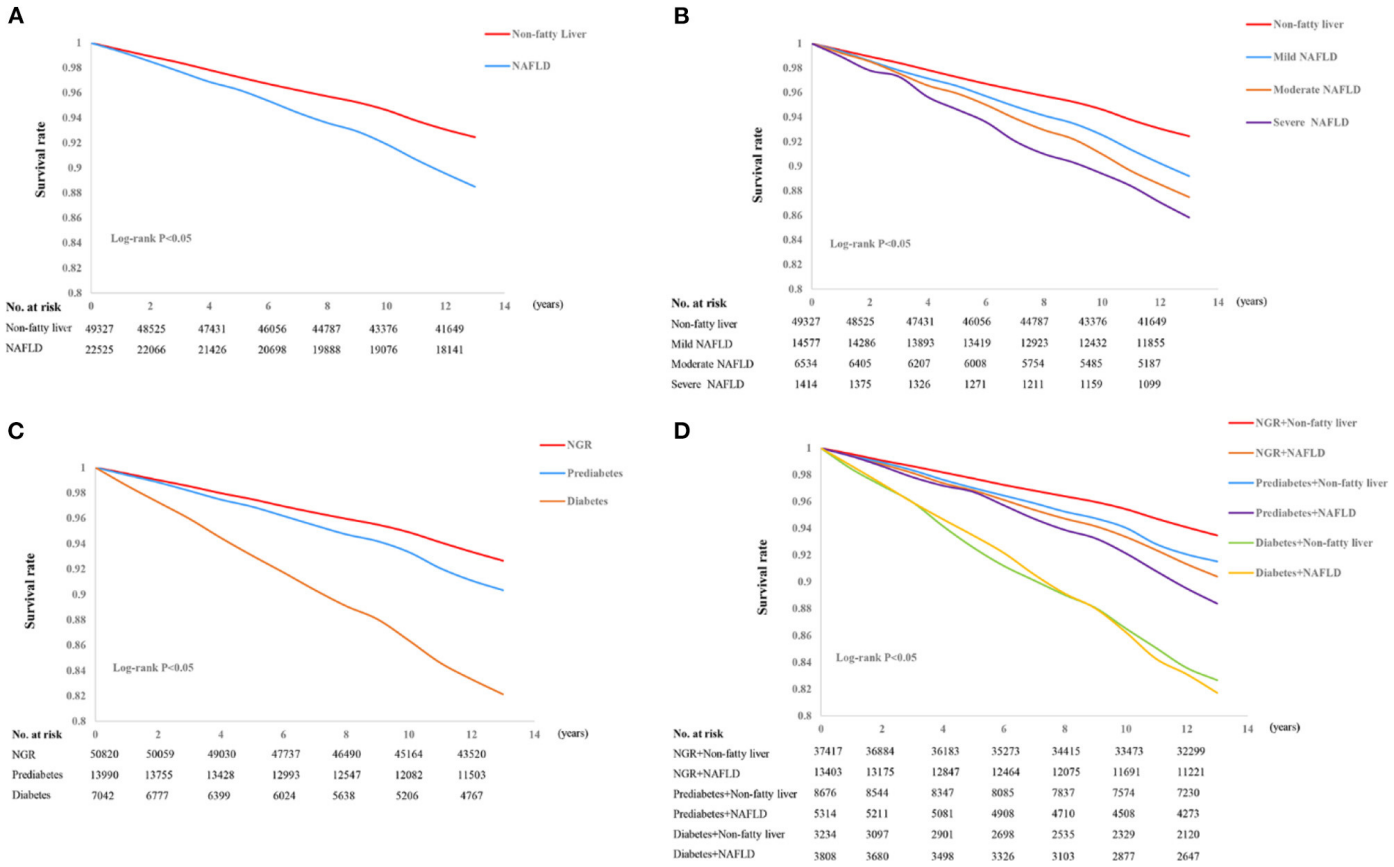
Kaplan-Meier curves for non-alcoholic fatty liver disease (NAFLD) **(A)**, the severity of NAFLD **(B)**, glucose control status **(C)**, and a combination of NAFLD and glucose metabolism **(D)**.

During a median follow-up period of 13.01 (0.64) years, 6,037 CVEs occurred (1,385 MIs and 4,652 strokes). Compared with participants without NAFLD, those with NAFLD had a 1.123-fold higher risk (95% CI 1.059–1.191) of CVEs after adjustment for confounding factors in the three models (Supplementary Table S1). As shown in Figure 2A, Kaplan-Meier analysis showed that participants with NAFLD had a lower event-free survival rate than those without (*P* < 0.001).

The participants were also allocated to four groups (no NAFLD, mild NAFLD, moderate NAFLD, or severe NAFLD) according to the severity of their NAFLD. Relative to participants without NAFLD, the HRs for participants with mild or moderate NAFLD were 1.449 (95% CI 1.365–1.539) and 1.694 (95% CI 1.568–1.831), respectively. Moreover, those with severe NAFLD were at a 1.957-fold higher risk of a CVE. This association remained significant after adjustment for potential confounders in the three models (all *P* < 0.001, Supplementary Table S1). Kaplan-Meier analysis showed that participants with more severe NAFLD were more likely to experience an event (*P* < 0.001, Figure 2B).

### Glucose Metabolism, NAFLD, and Cardiovascular Outcomes

The prevalence of CVEs in participants with pre-DM or DM group during the study were 9.2 and 16.4%, respectively. Univariate Cox regression analysis demonstrated that pre-DM and DM were associated with 1.333-fold (95% CI 1.251–1.420) and 2.605-fold (95% CI 2.438–2.784) higher risks of CVEs, respectively, compared with the NGR group. The adjustment for confounding factors in the three models only slightly attenuated these relationships (Supplementary Table S2).

Furthermore, we assessed the risk of CVE according to both the presence of NAFLD and glucose metabolism status. Univariate Cox proportional hazard regression analyses revealed that NAFLD was associated with a higher risk of CVE across the glucose metabolism status compared with the NGR and non-fatty liver group (HR 1.243, 95% CI 1.225–1.262, *P* for trend < 0.001, Table 2). Notably, NAFLD plus pre-DM and NAFLD plus DM were associated with 1.809- and 3.003-fold higher risks of CVEs, respectively (both *P* < 0.001). Adjustment for potential confounding factors in the three models did not abrogate these associations, with an adjusted HR of 1.267 (95% CI 1.151–1.395, *P* < 0.001) for the pre-DM plus NAFLD group and an adjusted HR of 1.829 (95% CI 1.666–2.008, *P* < 0.001) for the DM plus NAFLD group (Table 2). The risks of incident CVEs also remained significant after the exclusion of participants who experienced events within the first year of follow-up (Supplementary Table S3).

**Table 2:** Relationships of cardiovascular outcomes with non-alcoholic fatty liver disease and glucose metabolism status.

		**HR (95% CI)**							
**Variables**	**Events/subjects (6,037/71,852)**	**Crude model**	***P-*value**	**Model 1**	***P-*value**	**Model 2**	***P-*value**	**Model 3**	***P-*value**
**NGR**									
Non-fatty liver	(2,352/37,417)	Reference		Reference		Reference		Reference	
Nafld	(1,241/13,403)	1.493 (1.394–1.600)	<0.001	1.461 (1.364–1.565)	<0.001	1.298 (1.206–1.397)	<0.001	1.158 (1.075–1.247)	<0.001
**Pre-DM**									
Non-fatty liver	(706/8,676)	1.320 (1.214–1.436)	<0.001	1.192 (1.095–1.296)	<0.001	1.172 (1.077–1.275)	<0.001	1.100 (1.011–1.197)	0.0274
Nafld	(584/5,314)	1.809 (1.653–1.981)	<0.001	1.699 (1.552–1.860)	<0.001	1.498 (1.362–1.646)	<0.001	1.267 (1.151–1.395)	<0.001
**DM**
Non-fatty liver	(507/5,314)	2.864 (2.602–3.153)	<0.001	2.109 (1.915–2.322)	<0.001	2.052 (1.863–2.260)	<0.001	1.821 (1.653–2.007)	<0.001
Nafld	(647/3,808)	3.003 (2.753–3.276)	<0.001	2.536 (2.325–2.767)	<0.001	2.237 (2.042–2.451)	<0.001	1.829 (1.666–2.008)	<0.001
***P*** **for trend**		1.243 (1.225–1.262)	<0.001	1.190 (1.173–1.208)	<0.001	1.166 (1.148–1.184)	<0.001	1.126 (1.108–1.143)	<0.001

*BMI, body mass index; FBG, fasting blood glucose; HDL, high-density lipoprotein; HR, hazard ratio; hsCRP, high-sensitivity C-reactive protein; NAFLD, non-alcoholic fatty liver disease; TG, triglyceride*.

*Model 1 was adjusted for age and sex. Model 2 was adjusted for age, sex, physical activity, BMI (≥30, 25–29.9, 18.5–24.9, or <18.5 kg/m^2^), and smoking status. Model 3 was adjusted for age, sex, physical activity, BMI (≥30, 25–29.9, 18.5–24.9, or <18.5 kg/m^2^), smoking status, systolic blood pressure, hyperlipidemia, the use of lipid-lowering medication, HDL, hsCRP, and estimated glomerular filtration rate*.

Kaplan-Meier analysis with the log-rank test demonstrated that there was a significant difference in the cumulative event-free survival rates among the participants under different glucose metabolism status. Participants with DM were the most likely to experience an event (*P* < 0.001, Figure 2C). We also evaluated the prognostic utility of combining NAFLD and glucose metabolism status, and found that participants with both NAFLD and DM had the lowest cumulative event-free survival rate, followed by NAFLD plus pre-DM, compared with the reference group (non-NAFLD and NGR group, all comparisons *P* < 0.01, Figure 2D).

### Incremental Value of the NAFLD and Glucose Control Statuses Over Conventional Risk Factors for CVEs

In the predictive model comprising conventional risk factors, the C-statistic was 0.7331 (95% CI 0.7274–0.7388, Table 3). The addition of the presence of NAFLD to this model improved the C-statistic [ΔC-statistic 0.0008 (0.0004–0.0013), *P* < 0.001], and the addition of glucose metabolism status to the original model also significantly improved the C-statistic to 0.7396 (0.7340–0.7452) [ΔC-statistic 0.0065 (0.0051–0.0078), *P* < 0.001]. Finally, the incorporation of both the NAFLD and glucose metabolism status into the original model further increased the C-statistic to 0.7398 (0.7341–0.7454) [ΔC-statistic 0.0066 (0.0053–0.0080), *P* < 0.001].

**Table 3:** Incremental predictive values of non-alcoholic fatty liver disease and glucose metabolism status for cardiovascular outcomes.

**Models**	**C-statistic (95% CI)**	**ΔC-statistic (95% CI)**	***P-*value**
Original model	0.7331 (0.7274–0.7388)	-	-
Original model + NAFLD	0.7339 (0.7282–0.7396)	0.0008 (0.0004–0.0013)	<0.001
Original model + DM	0.7396 (0.7340–0.7452)	0.0065 (0.0051–0.0078)	<0.001
Original model + NAFLD + DM	0.7398 (0.7341–0.7454)	0.0066 (0.0053–0.0080)	<0.001

*BMI, body mass index; FBG, fasting blood glucose; HDL, high-density lipoprotein; HR, hazard ratio; hsCRP, high-sensitivity C-reactive protein; NAFLD, non-alcoholic fatty liver disease; TG, triglyceride*.

*Model 1 was adjusted for age and sex. Model 2 was adjusted for age, sex, physical activity, BMI (≥30, 25–29.9, 18.5–24.9, or <18.5 kg/m^2^), and smoking status. Model 3 was adjusted for age, sex, physical activity, BMI (≥30, 25–29.9, 18.5–24.9, or <18.5 kg/m^2^), smoking status, systolic blood pressure, hyperlipidemia, the use of lipid-lowering medication, HDL, hsCRP, and estimated glomerular filtration rate*.

## Discussion

In this community-based, observational prospective study of 71,852 people, we determined the effect of NAFLD on the incidence of CVEs in participants with NGR, pre-DM, or T2DM. Cox proportional hazard regression analyses demonstrated that participants with NAFLD were at 12.3% higher risk of a CVE after adjustment for potential confounding factors. Furthermore, more severe NAFLD was significantly associated with a higher risk of CVE. Notably, when they were categorized according to glucose metabolism status and the presence of NAFLD, we found that participants with pre-DM plus NAFLD and those with DM plus NAFLD had 1.267- and 1.829-fold higher risks of a CVE, respectively, than those with NGR and non-fatty liver disease. Furthermore, the addition of NAFLD to the original model improved its predictive efficiency, and the addition of a combination of the NAFLD and glucose metabolism status to the crude model further improved the prediction of CVE risk. Thus, the present findings suggest that patients with a combination of NAFLD and pre-DM or DM have a worse long-term clinical prognosis.

Accumulating evidence demonstrates that NAFLD is strongly associated with a higher risk of CVD, which is principally the result of a close interaction between NAFLD and other cardiometabolic disorders, including dyslipidemia, aberrant glucose metabolism, hypertension, and obesity (23, 24). Several studies have also shown that NAFLD contributes to the progression of subclinical atherosclerosis (increasing carotid artery intima-media thickness/plaques, arterial stiffness, and coronary artery calcification) and results in a higher prevalence of clinically apparent cardiovascular morbidity (24–26).

A number of epidemiological studies have shown that patients with NAFLD are more likely to experience ischemic stroke, MI, and cardiovascular-related mortality, independent of conventional risk factors (25, 27–29). The MESA study (Multi-Ethnic Study of Atherosclerosis) of 6,814 participants showed that NAFLD is associated with higher incidences of all-cause mortality and non-fatal CVEs (MI, resuscitated cardiac arrest, angina, or coronary revascularization) during a median follow-up period of 7.6 years (29). Similar results were obtained in a meta-analysis of 16 observational studies comprising 34,043 participants who were followed over a median follow-up period of 6.9 years. This showed that NAFLD confers a higher risk of non-fatal CVE and that participants with more severe NAFLD are more likely to experience a CVE (30). Finally, some other large studies have recently shown that NAFLD is independently associated with a higher incidence of MI, even in primary care populations. However, a recent population-based, retrospective, case-control study of 18 million European adults failed to show any significant association between NAFLD or non-alcoholic steatohepatitis (NASH) and the risk of experiencing an MI or stroke over a follow-up period of 2.1–5.5 years, after adjustment for established CVD risk factors (31). However, although this study was large and the participants were carefully matched, the lack of a significant association between NAFLD and CVEs may have been the results of misclassification bias, the design, and methodological issues (31). Thus, there is no consensus regarding whether the inclusion of NAFLD could improve the prediction of CVE (29). In the present large, population-based prospective study, we found that NAFLD confers a significantly higher long-term risk of a CVEs, independent of other risk factors. In addition, more severe NAFLD is associated with a higher risk of a CVE.

Recent studies have shown a reciprocal relationship between NAFLD and aberrant glucose metabolism that contributes to a higher risk of CVD. NAFLD is associated with fat accumulation in liver, aberrant metabolism, and the activation of inflammatory signals, which substantially accelerate the development of insulin resistance and glucose dysmetabolism (32, 33). Individuals with more severe NAFLD are more likely to have prediabetes or diabetes (12, 34). NAFLD is independently associated with a two-to-five-fold higher risk of T2DM, even after adjustment for other, potentially confounding risk factors (35). A cohort study of 88 participants with biopsy-confirmed NAFLD showed that 69 developed impaired glucose tolerance or DM during a mean follow-up period of 13.7 years. In addition, participants with progressive liver fibrosis experienced more serious insulin resistance (36), and those with cirrhosis also had a higher incidence of T2DM than those with mild or moderate fibrosis (37).

Conversely, insulin resistance and diabetes have been shown to promote the progression of hepatic steatosis to NASH and biopsy-confirmed fibrosis (38). It has been reported that patients with T2DM have a significantly higher incidence of NAFLD, ranging from 41.6 to 86% (32). Further studies based on the histology of liver biopsies have revealed that patients with DM are at a higher risk of developing severe NAFLD than those without DM, with a prevalence of NASH of up to 80% and a prevalence of advanced fibrosis of up to 30% in all patients (34, 36). Moreover, the presence of NAFLD in individuals with DM not only results in poor glucose control, but also predisposes toward microvascular and macrovascular disorders, such as diabetic nephropathy, diabetic retinopathy, cardiovascular disease, and stroke, as well as all-cause mortality (38, 39). Specifically, the presence of NAFLD in patients with diabetes confers a two-fold additional risk of overall mortality compared with those without NAFLD (34).

Targher et al. (40) found a significant value of NAFLD for the prediction of CVE in patients with type 1 diabetes, and they also (39) found that NAFLD is associated with a higher risk of cardiovascular events (cardiovascular death, non-fatal MI, late coronary revascularization, non-fatal stroke, or hospitalization for heart failure) in patients with T2DM and suspected coronary artery disease. A recent cross-sectional study also showed that NAFLD is linked to a higher prevalence of cardiovascular risk in patients with prediabetes or diabetes (41). However, whether NAFLD increases the prognostic value of the presence of prediabetes or diabetes for CVE in the long term remains to be explored. In the present study, we analyzed the prognosis of NAFLD according to glucose metabolic status. The main findings were that patients with NAFLD and pre-DM or DM are at 1.267- and 1.829-fold higher risks of CVE after adjustment for other potential confounding factors, which confirms the importance of NAFLD in patients with pre-DM or DM.

The present study had several limitations. First, it was an observational study, and therefore may have been influenced by the non-random assignment of exposure. Second, the criteria for the diagnosis of NAFLD was based on ultrasonographic examination, instead of on liver biopsy; therefore, we could not assess the relationship between liver fibrosis and subsequent CVE. In addition, DM was defined using FBG concentration, rather than hemoglobin A1c, and therefore the relationship between long-term glucose status and CVE could not be investigated. Third, we recruited participants from a community-based study, which may limit the applicability and generalizability of the results. Fourth, a stratified analysis among the stages of glucose metabolism can only indicate that patients with NAFLD at different stages of study have different risk of CVE, there might be modification effect, which requires further exploration through quantitative analysis.

## Conclusions

In this population-based prospective study, we have shown that more severe NAFLD is associated with a higher risk of CVE. Furthermore, the presence of both NAFLD and pre-DM was associated with a worse prognosis, and the presence of both NAFLD and DM was associated with the highest risk, indicating that the consideration of a diagnosis of NAFLD may aid the cardiovascular risk stratification of patients with pre-DM or DM.

## Data Availability Statement

The raw data supporting the conclusions of this article will be made available by the authors, without undue reservation.

## Ethics Statement

The study complied with the principles of the Declaration of Helsinki and was approved by the Ethics Committee of the Kailuan Medical Group, Kailuan Group. All the participants gave their written informed consent.

## Author Contributions

S-LW and JC planned the study. Q-RS and S-LL conducted a survey, analyzed the data, and wrote the article. S-HC and Y-GB contributed to the drafting. All authors read and approved the final manuscript.

## Funding

This work was supported by CAMS Innovation Fund for Medical Sciences (No. 2021-1-I2M-007), National Natural Science Foundation of China (Nos. 81825002 and 81800367), Beijing Outstanding Young Scientist Program (No. BJJWZYJH01201910023029), Capital Clinical Diagnosis and Treatment Technology Research and Demonstration Application Project of Beijing Science and Technology Commission (No. Z191100006619106), and AI+ Health Collaborative Innovation Cultivation Project of Beijing Science and Technology Commission (No. Z201100005620006).

## Conflict of Interest

The authors declare that the research was conducted in the absence of any commercial or financial relationships that could be construed as a potential conflict of interest.

## Publisher's Note

All claims expressed in this article are solely those of the authors and do not necessarily represent those of their affiliated organizations, or those of the publisher, the editors and the reviewers. Any product that may be evaluated in this article, or claim that may be made by its manufacturer, is not guaranteed or endorsed by the publisher.

## Abbreviations

NAFLD: non-alcoholic fatty liver disease
HBsAg: hepatitis B surface antigen
FBG: fasting blood glucose
NGR: normal glucose regulation
DM: diabetes mellitus
pre-DM: prediabetes
MI: myocardial infarction
HS: hemorrhagic stroke
IS: ischemic stroke
CR: creatinine
UA: uric acid
TC: total cholesterol
LDL-C: low-density lipoprotein-cholesterol
HDL-C: high-density lipoprotein-cholesterol
TG: triglyceride
eGFR: estimated glomerular filtration rate
BP: blood pressure
BMI: body mass index
HR: hazard ratio
CI: confidence interval
SBP: systolic blood pressure
hsCRP: high-sensitivity C-reactive protein
NASH: non-alcoholic steatohepatitis.
