# Supplementary material for: Non-alcoholic Fatty Liver Disease Is Associated With Cardiovascular Outcomes in Subjects With Prediabetes and Diabetes: A Prospective Community-Based Cohort Study
Source: Front Cardiovasc Med. 2022 Apr 26;9:889597. doi: 10.3389/fcvm.2022.889597 (PMC9086774; doi:10.3389/fcvm.2022.889597)
Supplement: Supplementary file 1 [file Data_Sheet_1.docx]

***SUPPLEMENTARY DATA***

**Supplementary Table S1.** Relationship of non-alcoholic fatty liver disease with cardiovascular outcomes, on the basis of univariate and multivariate survival analysis

|  |  | **HR (95% CI)** | | | | | | | |
| --- | --- | --- | --- | --- | --- | --- | --- | --- | --- |
| **Variables** | **Events/subjects (6037/71852)** | **Crude model** | **P value** | **model 1** | **P value** | **model 2** | **P value** | **model 3** | **P value** |
| **The presence of NAFLD** | | | | | | | | | |
| **Nonfatty liver** | (3565/49327) | Reference |  | Reference |  | Reference |  | Reference |  |
| **Nafld** | (2472/22525) | 1.551 (1.474-1.633) | <0.001 | 1.527 (1.451-1.608) | <0.001 | 1.345 (1.270-1.424) | <0.001 | 1.123 (1.059-1.191) | <0.001 |
| **The severity of NAFLD** | | | | | | | | | |
| **Nonfatty liver** | (3565/49327) | reference |  | reference |  | reference |  | reference |  |
| **Mild NAFLD** | (1501/14577) | 1.449 (1.365-1.539) | <0.001 | 1.423 (1.340-1.511) | <0.001 | 1.282 (1.203-1.367) | <0.001 | 1.104 (1.035-1.179) | <0.001 |
| **Moderate NAFLD** | (780/6534) | 1.694 (1.568-1.831) | <0.001 | 1.663 (1.539-1.797) | <0.001 | 1.444 (1.329-1.570) | <0.001 | 1.149 (1.055-1.251) | <0.001 |
| **Severe NAFLD** | (191/1414) | 1.957 (1.692-2.264) | <0.001 | 2.020 (1.746-2.337) | <0.001 | 1.684 (1.446-1.962) | <0.001 | 1.235 (1.059-1.441) | <0.001 |

BMI, body mass index; FBG, fasting blood glucose; HDL, high-density lipoprotein; HR, hazard ratio; hsCRP, high-sensitivity C-reactive protein; NAFLD, non-alcoholic fatty liver disease; TG, triglyceride. Model 1 was adjusted for age and sex. Model 2 was adjusted for age, sex, physical activity, BMI (≥30, 25–29.9, 18.5–24.9, or <18.5 mg/m2), and smoking status. Model 3 was adjusted for age, sex, physical activity, BMI (≥30, 25–29.9, 18.5–24.9, or <18.5 kg/m2), smoking status, systolic blood pressure, hyperlipidemia, the use of lipid-lowering medication, HDL, hsCRP, and estimated glomerular filtration rate.

**Supplementary Table S2.** Relationship of glucose control status with cardiovascular outcomes, on the basis of univariate and multivariate survival analysis

|  |  | **HR (95% CI)** | | | | | | | |
| --- | --- | --- | --- | --- | --- | --- | --- | --- | --- |
| **Diabetic status** | **Events/subjects (****6037/71852)** | **Crude model** | **P value** | **model 1** | **P value** | **model 2** | **P value** | **model 3** | **P value** |
| **NGR** | (3593/50820) | Reference |  | Reference |  | Reference |  | Reference |  |
| **Pre-DM** | (1290/13990) | 1.333 (1.251-1.420) | <0.001 | 1.228 (1.153-1.309) | <0.001 | 1.187 (1.113-1.265) | <0.001 | 1.101 (1.032-1.174) | <0.001 |
| **DM** | (1154/7042) | 2.605 (2.438-2.784) | <0.001 | 2.079 (1.945-2.222) | <0.001 | 1.952 (1.825-2.088) | <0.001 | 1.689 (1.577-1.809) | <0.001 |

BMI, body mass index; FBG, fasting blood glucose; HDL, high-density lipoprotein; HR, hazard ratio; hsCRP, high-sensitivity C-reactive protein; NAFLD, non-alcoholic fatty liver disease; TG, triglyceride. Model 1 was adjusted for age and sex. Model 2 was adjusted for age, sex, physical activity, BMI (≥30, 25–29.9, 18.5–24.9, or <18.5 mg/m2), and smoking status. Model 3 was adjusted for age, sex, physical activity, BMI (≥30, 25–29.9, 18.5–24.9, or <18.5 kg/m2), smoking status, systolic blood pressure, hyperlipidemia, the use of lipid-lowering medication, HDL, hsCRP, and estimated glomerular filtration rate.

**Supplementary Table S3.** Relationship of non-alcoholic fatty liver disease with cardiovascular outcomes in participants categorized according to glucose metabolism status, after the exclusion of those who experienced a CVD event within the first year of follow-up (N = 71,257)

|  | **HR (95% CI)** | | | | | | | |
| --- | --- | --- | --- | --- | --- | --- | --- | --- |
| **Variables** | **Crude model** | **P value** | **model 1** | **P value** | **model 2** | **P value** | **model 3** | **P value** |
| **NGR** |  | | | | | | | |
| Nonfatty liver | ref |  | ref |  | ref |  | ref |  |
| Nafld | 1.510 (1.406-1.621) | <0.001 | 1.474 (1.372-1.582) | <0.001 | 1.310 (1.214-1.414) | <0.001 | 1.169 (1.083-1.263) | <0.001 |
| **Pre-DM** |  | | | | | | | |
| Nonfatty liver | 1.319 (1.209-1.440) | <0.001 | 1.190 (1.090-1.298) | <0.001 | 1.172 (1.074-1.280) | <0.001 | 1.099 (1.007-1.200) | 0.0348 |
| Nafld | 1.845 (1.680-2.025) | <0.001 | 1.728 (1.574-1.897) | <0.001 | 1.523 (1.382-1.680) | <0.001 | 1.289 (1.168-1.423) | <0.001 |
| **DM** |  | | | | | | | |
| Nonfatty liver | 2.806 (2.537-3.103) | <0.001 | 2.079 (1.879-2.301) | <0.001 | 2.027(1.832-2.243) | <0.001 | 1.799 (1.625-1.992) | <0.001 |
| Nafld | 3.010 (2.750-3.295) | <0.001 | 2.548 (2.327-2.789) | <0.001 | 2.249 (2.046-2.473) | <0.001 | 1.839 (1.669-2.026) | <0.001 |

BMI, body mass index; FBG, fasting blood glucose; HDL, high-density lipoprotein; HR, hazard ratio; hsCRP, high-sensitivity C-reactive protein; NAFLD, non-alcoholic fatty liver disease; TG, triglyceride. Model 1 was adjusted for age and sex. Model 2 was adjusted for age, sex, physical activity, BMI (≥30, 25–29.9, 18.5–24.9, or <18.5 mg/m2), and smoking status. Model 3 was adjusted for age, sex, physical activity, BMI (≥30, 25–29.9, 18.5–24.9, or <18.5 kg/m2), smoking status, systolic blood pressure, hyperlipidemia, the use of lipid-lowering medication, HDL, hsCRP, and estimated glomerular filtration rate.
